# Sentinel lymph node biopsy using single-agent mapping tracer (blue dye) after neoadjuvant chemotherapy in a Brazilian cohort of breast cancer patients. Real world evidence

**DOI:** 10.1590/ACB360608

**Published:** 2021-07-02

**Authors:** Heloisa Magda Resende, Martina Lichtenfels, Igor Camargo Soares, Angélica Araújo Cortines Laxe Renó, Ana Paula Cunha, Pedro Gustavo Falcão, Carolina Soares Pimentel Pieroni, Biazi Ricieri de Assis, Paola Cardoso, Pedro Henrique Adário Marassi, Rafael dos Santos Reis

**Affiliations:** 1 PhD. Department of Oncology – Hospital Jardim Amália, and Centro Universitário de Volta Redonda – Volta Redonda (RJ), Brazil.; 2 PhD. Breast Cancer Center – Hospital São Lucas – Porto Alegre (RS), Brazil.; 3 MD. Department of Mastology, Hospital Jardim Amália – Volta Redonda (RJ), Brazil.; 4 MD. Laboratório Falcão – Volta Redonda (RJ), Brazil.; 5 Pharmacist. Department of Oncology – Hospital Jardim Amália – Volta Redonda (RJ), Brazil.; 6 MD. Department of Oncology – Hospital Jardim Amália, and Centro Universitário de Volta Redonda – Volta Redonda (RJ), Brazil.; 7 Graduate student. School of Medicine – Centro Universitário de Volta Redonda (Unifoa) – Volta Redonda (RJ), Brazil.

**Keywords:** Sentinel Lymph Node Biopsy, Lymph Node Excision, Neoadjuvant Therapy, Breast Neoplasms

## Abstract

**Purpose:**

To reduce false-negative rates (FNR) in sentinel lymph node biopsy (SLNB) of clinically positive (cN+) axilla in patients undergoing neoadjuvant chemotherapy (NAC). The removal of three or more lymph nodes with dual-tracer mapping including a radioisotope was used. However, in the Brazilian Unified Health System, the radioisotope tracer is not feasible in some hospitals. We conducted a cross-sectional study to evaluate the detection rate of sentinel lymph node (SLN) in patients who converted from cN+ to ycN0 after NAC using blue dye as a single-agent mapping tracer.

**Methods:**

During the period of March 2018 to September 2019, 34 patients who underwent NAC with cN+ who converted to ycN0 were enrolled in the study. The SLNB was performed using blue dye as a single-agent mapping followed by axillary lymph node dissection (ALND).

**Results:**

The detection rate of sentinel lymph node was of 85.3%, being SLNB not possible for five patients (14.7%), due to fibrosis. The mean number of removed SLN was 2.5.

**Conclusions:**

The use of blue dye as a single-agent mapping tracer demonstrated an acceptable detection rate of 85.3%. Although the FNR was possible to be determined, the small sample size might overestimate this rate. The removal of three or more lymph nodes with single-agent mapping tracer might be indicated for breast cancer patients who converted to ycN0 after NAC in the Brazilian health public services, in which radioisotope tracer is not suitable.

## Introduction

Sentinel lymph node biopsy (SLNB) has been the standard care for breast cancer patients with clinically node-negative axilla (cN0) since Morrow *et al*.^1^ demonstrated no statistically significant difference in locoregional recurrence rates using SLNB or axillary lymph node dissection (ALND) in the Z011 trial. The de-escalation of breast cancer surgery has been a trend in mammary oncology, moving the bar to less morbid procedures for breast cancer patients^2^-^3^. Neoadjuvant chemotherapy (NAC) is frequently indicated for breast cancer patients with clinically node-positive (cN+) axilla, but SLNB has been done only for selected patients^4^.

The tumor downstaging is represented by breast and/or axilla decreasing tumor, with possibility to revert a cN+ axilla to clinically node-negative (ycN0) axilla, which is the requirement for SLNB after NAC^5^-^6^. Although expressive clinical trials addressed the use of SLNB after NAC, it remains controversial^4^,^7^-^9^. The main concern about this procedure is the false-negative rate (FNR), which could leave residual disease in the axilla. Besides that, the unknown residual disease in the axilla would prevent patients from being tailored to new chemotherapy protocols, which would improve their survival^10^,^11^.

The use of dual-agent mapping tracer and removal of more than one sentinel lymph node (SLN) is a recommended strategy to decrease FNR^12^. In Brazil, there are some hospitals in the Unified Health System (Sistema Único de Saúde*—*SUS) where dual-agent mapping tracer is not feasible, which makes SLNB after NAC particularly challenging. It is necessary to establish requirements for the acceptance of SLNB after NAC in those patients managed in the SUS who converted from cN+ to ycN0 axilla and presented SLN negative in the freezing technique during surgery.

We conducted a cross-sectional study to demonstrate the detection rate of SLN using single-agent mapping tracer (blue dye) in breast cancer patients treated in one hospital through the SUS.

## Methods

This study was conducted at Hospital Jardim Amália, in the Mastology Department, and approved by the Ethics Committee of the Fundação Oswaldo Aranha (CoEPS), number 84059818.5.0000.5237.

Thirty-four patients with pathologically confirmed invasive breast cancer T1 through T4, N1 through N2, M0 who converted to clinically node-negative axilla after NAC were enrolled in the study from March 2018 to September 2019. Axillary staging was performed by physical exam by two examiners and based on ultrasound exam. Patients were staging according to the eighth edition of the American Joint Committee on Cancer Staging Manual. During surgical procedure, all the patients underwent SLNB (the sentinel lymph nodes were analyzed by freezing technique) followed by ALND.

All patients signed the informed consent form at the end of NAC and before the surgical procedure. Clinicopathological characteristics of patients are listed in Table 1.

**Table 1:** Clinicopathological characteristics of the 34 patients.

CLINICOPATHOLOGICAL CHARACTERISTICS		
Total of patients = 34	N	%
Age (years old)Mean patient age = 54		
≤ 40	3	8.8
41 – 50	12	35.2
51 – 60	9	26.4
≥ 61	10	29.4
**Clinical category at diagnosis**		
T_2_N_1_	18	53
T_2_N_2_	8	23.5
T_3_N_1_	3	8.8
T_3_N_2_	3	8.8
T_1_N_1_	1	3
T_4_N_1_	1	3
**Subtype Molecular**		
Luminal HER-2 positive	7	20.6
Luminal HER-2 negative	17	50
HER-2 positive	3	8.8
Triple negative	7	20.6
**Histology**		
Ductal	31	91.2
Mixed (ductal and lobular)	3	8.8

### Chemotherapy regimen

NAC was performed according to the institution guideline: four cycles of intravenous (i.v.) doxorubicin60 mg/m^2^ (generic drug) plus cyclophosphamide 600 mg/m^2^i.v. (generic drug) followed by weekly i.v. paclitaxel80 mg/m^2^ (generic drug) for 12 weeks, or four-six cycles of docetaxel 75 mg/m^2^ (generic drug) plus cyclophosphamide600 mg/m^2^ (generic drug). Trastuzumab (trastuzumab reference Herceptin®) was added for HER2 positive patients during the taxane phase. After surgery, 13 cycles of i.v. trastuzumab (trastuzumab reference Herceptin®) 6 mg per kilogram of body weight was added for HER2 positive patients.

### Surgical procedures

Patients who converted to clinically node-negative axilla ycN0 after NAC confirmed by physical exam (two examiners) and ultrasound exam underwent SLNB planning to remove at least two lymph nodes. These procedures were performed with the injection of blue dye. The mapping agent is taken up by the breast lymphatics as they travel to the axillary nodes. The blue dye stains the lymphatic channels and accumulates in the lymph nodes. Additionally, the axilla is carefully palpated, and abnormal lymph nodes are identified and removed.

The removed lymph nodes were submitted to pathological analyses by freezing technique during perioperative procedure and followed by histopathologic analysis in paraffin block. Both the negative and positive SLNs groups of patients underwent the ALND. Treatment characteristics are shown in Table 2.

**Table 2:** Treatment characteristics of the 34 patients.

TREATMENT		
Total of patients = 34	N	%
Time between neoadjuvant chemotherapy and surgery		
≤ 8 weeks	16	47.1
> 8 weeks	18	52.9
**Chemotherapy protocol**		
Anthracycline and taxane based	32	94.1
Taxane based	2	5.9
**Findings on axilla and breast after chemotherapy**		
No palpable adenopathy	34	100
No palpable tumor in breast	20	58.9
**Breast surgery after chemotherapy**		
Conservative surgery	17	50
Total mastectomy	17	50

## Results

The mean patient age was 54 years old (35-85 years), three patients (8.8%) were younger than 40 years old and 10 patients (29.4%) older than 60 years old (Table 1).

Clinical stage cT2N1 was the most frequent (n=18, 53%) in our population, followed by 23.4% of cT2N2 (n=8), 8.8% of cT3N1 (n=3), and 8.8% of cT3N2 (n=3). Only 3% of the patients presented clinical stages cT1N1 and 3% cT4N1. Thirty-one patients (91.2%) presented invasive ductal carcinoma, and three (8.8%) presented mixed invasive ductal and lobular carcinoma. Most of the patients presented hormonal receptor positive/HER2 negative tumors (50%).

The most used chemotherapy regimen was anthracycline and taxane based, with 32 patients (94.1%), included in the protocol, and only two (5.9%) patients received taxane plus cyclophosphamide regimen. The time between the end of NAC and surgery was less than eight weeks for 16 patients (47.1%) and more than eight weeks for 18 patients (52.9%). From the 34 patients that underwent NAC and converted from clinically positive axilla to clinically negative axilla, 20 (58.8%) obtained clinical complete response in the breast and axilla (Table 2).

**Figure 1:**
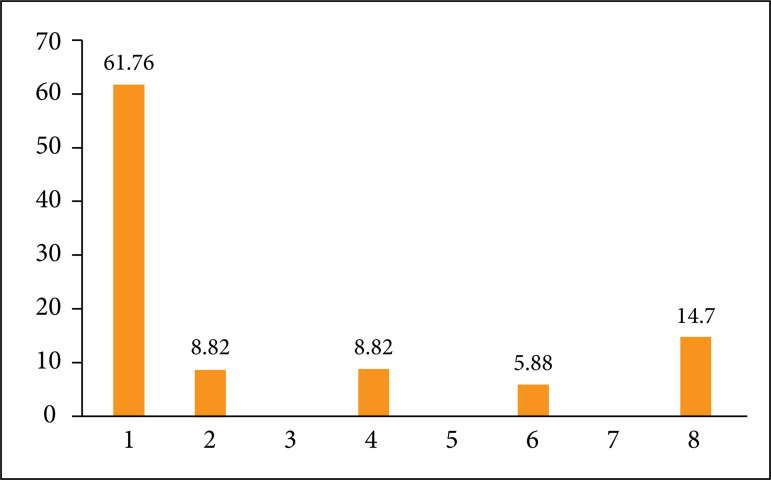
Concordance between sentinel lymph node biopsy and axillary lymph node dissection.

In the study protocol, it was planned to perform SLNB followed by ALND for all the patients. However, during operative procedure, five patients (14.3%) presented axillary fibrosis, which make impossible to perform the SLNB. Therefore, these five patients underwent ALND without SLNB, yielding 85.7% of SLN detection rate. These five patients presented positive lymph nodes by ALND. Among the 29 patients who submitted to SLNB, one had only one lymph node removed, 13 patients had two lymph nodes removed, seven patients had three lymph nodes removed, four patients had four, three patients had five lymph nodes removed, and one patient had six (Table 3). The mean number of sentinel lymph nodes removed was 2.5. Twenty-one patients had negative SLNs in SLNB and also in ALND, six patients had positive SLN in SLNB, having three of them positive lymph nodes in ALND sampling, and the other three patients (10.3%) had negative lymph nodes in ALND sampling. Two patients had negative SLN in SLNB and positive lymph nodes in ALND sampling (Fig. 1).

**Table 3:** Number of removed sentinel lymph node (SLN) by patient.

SLN removed (N)	Patients (N)	Patients (%)
1	1	2.94
2	13	38.24
3	7	12.94
4	4	7.62
5	3	6.67
6	1	2.94
ALND alone	5	14.71

ALND: axillary lymph node dissection.

Thus, amongst eight patients with pathologically positive lymph nodes who underwent SLNB and ALND, two had negative SLN. Although it would be possible to calculate FNR (25%), the small sample size might overestimate this rate.

## Discussion

Contemporary clinical trials and meta-analyses recommend the SLN mapping with dual-agent including a radioisotope after NAC^7^-^9^,^12^, but that is not suitable in some Brazilian public hospitals, which requires that the doctors find the ideal cohort of patients to receive SLNB with single-agent mapping tracer to spare them from ALND.

The incorporation of SLNB after NAC is really advantageous in order to decrease morbidity. NAC is becoming increasingly important to enhance the used of conservative surgery decreasing morbidity^5^, and tailoring chemotherapy strategies, which means, if the breast and axilla have no residual disease after NAC (complete pathological response–pCR), the adjuvant chemotherapy regimen is the same as previously planned^10^,^11^.

On the order hand, in node-positive axilla or residual disease in breast after NAC, it is desirable to include chemotherapy courses as capecitabine for triple negative tumors and T-DM1 for HER2 positive breast cancer^10^,^11^.

Many researchers have been investigating the use of SLNB in patients who convert a clinically node-positive cN+ to a ycN0 axilla after NAC in large trials such ACOSOG Z1071, Sentinel Neoadjuvant (SENTINA), and SN FNAC^7^-^9^. Regardless of that, SLNB after NAC remains controversial^4^. Although we need to incorporate SLNB after NAC, there are still concerns regarding detection rate and FNR, which might be related to the learning curve of the surgery team, number of removed lymph nodes, dual-agent versus single-agent mapping tracer and clinicopathological characteristics of the patients.

Furthermore, we are particularly worried with Brazil, where 63% of breast cancer patients have been treated by the SUS^13^, but this system is heterogeneous and presents several coverage discrepancies according to distinct geographic areas^14^,^15^. Restrictions regarding the use of dual-agent mapping tracer in some Brazilian public SUS hospitals, including ours, show the underfunding in the public health system.

According to Frasson *et al*.^16^, 11.3% of breast surgeons who work into SUS have claimed that the SLNB is not suitable in such hospitals, and we do not know whether the other 88.7% have the single-agent or dual-agent mapping lymph nodes tracer^17^. In our hospital, we only have blue dye (patent blue) available, and we conducted this cross-sectional study to demonstrate the detection rate of SLN using it to reflect a real-world evidence. Our SLN detection rate was of 85.3%, which would be acceptable, comparing with National Surgical Adjuvant Breast and Bowel Project (NSABP) B-27, with identification and removal of a sentinel lymph node of 84.8%, SENTINA arm C (80.1%), ACOSOG 1071 (92.7%), and Aguiar (85.3%)^5^-^8^,^18^. Although it would be possible to calculate FNR in our study (25%), the small sample size might overestimate this rate. The mean number of removed lymph nodes was 2.5 (0-6), also slightly lower than the recommended by some investigators which demonstrated that removal of at least three lymph nodes is related with lower FNR^12^.

The small number of enrolled patients does not permit us to define conclusions, and the current cross-sectional study could be a hypothesis-generator study which needs to be confirmed by other studies using single-agent blue dye mapping tracer in breast cancer surgery with patients cT1 through 4, N1 through N2 who converted from cN+ to ycN0 after NAC.

## Conclusions

This cross-sectional study yielded a real-word evidence in a small Brazilian cohort of breast cancer patients receiving NAC in the SUS scenario. The use of blue dye as a single-agent mapping tracer demonstrated an acceptable SLN detection rate of 85.3%. However, due to the small sample size, it was not possible to calculate FNR in patients cN+ who converted to ycN0 after NAC. The removal of three or more lymph nodes in services in which radioisotope tracer is not suitable might help to overcome the lack of such mapping tracer in patients who obtained clinically negative axilla after NAC. The current cross-sectional study could be a hypothesis-generator study which needs to be confirmed by new studies using single-agent blue dye mapping tracer in patients who converted from cN+ to ycN0 after NAC.

## References

[1] Giuliano A, McCall L, Beitsch P, Withworth PW, Blumecranz P, Leitch AM, Saha S, Hunt K, Morrow M, Ballman K. (2010). Locoregional recurrence after sentinel lymph node dissection with
or without axillary dissection in patients with sentinel lymph node
metastases: The American College of Surgeons Oncology Group Z0011 randomized
trial. Ann Surg.

[2] Morrow M, Winer EP. (2020). De-escalating breast cancer surgery – where is the tipping
point?. JAMA Oncol.

[3] Wang T, Baskin AS, Dossett LA. (2020). Deimplementation of the choosing wisely recommendations for
low-value breast cancer surgery a systematic review. JAMA Surg.

[4] Vracken Peeters MTFD (2019). Management of the axilla after neoadjuvant chemotherapy for
breast cancer. Br J Surg.

[5] Bonadonna G, Veronesi U, Brambilla C, Ferrari L, Luini A, Greco M, Bartoli C, Yoldi G, Zucali R, Rilke F, Andreola S, Silvestrini R, Di Fronzo, Valagussa P (1990). Primary chemotherapy to avoid mastectomy in tumors with diameters
of three centimeters or more. J Natl Cancer Inst.

[6] Mamounas EP, Brown A, Anderson S, Smith R, Julian T, Miller B, Bear HD, Caldwell C, Walker A, Mikkelson W, Stauffer JS, Robidoux A, Theoret H, Soran A, Fisher B, Wickerham DL, Wolmark N (2005). Sentinel node biopsy after neoadjuvant chemotherapy in breast
cancer: results from national surgical adjuvant breast and bowel project
protocol B-27. J Clin Oncol.

[7] Boughey J, Suman V, Mittendorf E, Ahrendt GM, Wilke L, Taback B, Leitch A, Kuerer H, Bowling M, Flippo-Morton T, Byrd D, Ollila D, Julian T, Laughlin S, McCall L, Symmans F, Le-Petross H, Haffty B, Buchholz T, Nelson H, Hunt K (2013). Sentinel lymph node surgery after neoadjuvant chemotherapy in
patients with node-positive breast cancer the ACOSOG Z1071 (Alliance)
clinical trial. JAMA.

[8] Kuehn T, Bauerfeind I, Fehm T, Fleige B, Hausschild M, Helms G, Lebeau A, Liedtke C, von Minckwitz, Nekljudova V, Schmatloch S, Schrenk P, Staebler A, Untch M. (2013). Sentinel-lymph-node biopsy in patients with breast cancer before
and after neoadjuvant chemotherapy (SENTINA): a prospective, multicentre
cohort study. Lancet Oncol.

[9] Boileau J, Poirier B, Basik M, Holloway C, Gaboury L, Sideris L, Meterissian S, Arnaout A, Brackstone M, McCready D, Karp S, Trop I, Lisbona A, Wright F, Rami J, Provencher L, Patocskai E, Omeroglu A, Robidoux A (2015). Sentinel node biopsy after neoadjuvant chemotherapy in
biopsy-proven node-positive breast cancer: The SN FNAC study. J Clin Oncol.

[10] Zujewski JA, Rubinstein L. (2017). CREATE-X a role for capecitabine in early-stage breast cancer: an
analysis of available data. NPJ Breast Cancer.

[11] Minckwitz G., Huang C, Mano M, Loibl S, Mamounas E, Untch M, Wolmark N, Rastogi P, Schneeweiss A, Redondo A, Fischer HH, Jacot W, Conlin A, Wapnir I, Jackish C, DiGiovanna M, Fasching P, Crown J, Wülfing P, Shao Z, Caremoli ER, Wu H, Lam LH, Tesarowski D, Smitt M, Douthwaite H, Singel SM, Geyer C (2019). Trastuzumab emtansine for residual invasive HER2-positive breast
cancer. N Engl J Med.

[12] Barros A, Andrade D (2020). Extended sentinel node biopsy in breast cancer patients who
achieve complete nodal response with neoadjuvant
chemotherapy. Eur J Breast Health.

[13] Rosa DD, Bines J, Werutsky G, Barrios CH, Cronemberger E, Queiroz GS, Lima V, Freitas-Júnior R, Couto JO, Emerenciano K, Resende H, Crocamo S, Reinert T, Van Eyil, Nerón Y, Dybal V, Lazaretti N, Costamilan RC, Andrade DAP, Mathias C, Vacaro GW, Borges G, Morelle A, Caleffi M, Sampaio C, Mano M, Zaffaroni F, Jesus RG, Simon SD (2020). The impact of sociodemographic factors and health insurance
coverage in the diagnosis and clinicopathological characteristics of breast
cancer in Brazil: AMAZONA III study (GBECAM 0115). Breast Cancer Res Treat.

[14] Fernandes GS, Sternberg C, Lopes G, Chammas R, Gifoni MAC, Gil RA, Araujo DV. (2018). The use of biosimilar medicines in oncology - position statement
of the Brazilian Society of Clinical Oncology (SBOC). Braz J Med Biol Res.

[15] Castro MC, Massuda A, Almeida G, Menezes N, Andrade M, Noronha K, Rocha R, Macinko J, Hone T, Tasca R, Giovanella L, Malik AM, Werneck H, Fachini L, Atun R (2019). Brazil’s unified health system : the first 30 years and perspects
for the future. Lancet.

[16] Frasson AL, Resende HM, Lichtenfels M, Barbosa F, Souza ABA, Miranda I, Facolne AB (2020). Axillary management for patients with breast cancer after
neoadjuvant chemotherapy: results of a survey among Brazilian breast
surgeons. J Surg Oncol.

[17] Xing Y, Foy M, Cox DD, Kuerer HM, Hunt KK, Cormier JN. (2006). Meta-analysis of sentinel lymph node biopsy after preoperative
chemotherapy in patients with breast cancer. Br J Surg.

[18] Aguiar PHW, Pinheiro LGP, Mota RMS, Margotti NHG, Rocha JIX (2012). Sentinel lymph node biopsy in patients with locally advanced
breast cancer after neoadjuvant chemotherapy. Acta Cir Bras.

